# Rice Straw-Derived Magnetic Hydrothermal Carbon Accelerates Anaerobic Azo Dye Biodegradation Through Enhanced Interspecies Electron Transfer

**DOI:** 10.3390/biology15120896

**Published:** 2026-06-07

**Authors:** Lei Ma, Yong Tian, Xinyu Che, Wentao Ouyang, Ran Bi, Min Zhao, Daizong Cui

**Affiliations:** 1 College of Life Science, Northeast Forestry University, Harbin 150040, China; 15684195808@163.com (L.M.);; 2 Key Laboratory for Enzyme and Enzyme-Like Material Engineering of Heilongjiang, Harbin 150040, China

**Keywords:** Fe_3_O_4_@hydrothermal carbon, “anaerobic sludge–material” combining system, azo dye decolorization, sludge electroactivity and structure, material-mediated electron transfer

## Abstract

In the present study, Fe_3_O_4_@hydrothermal carbon was prepared successfully using rice straw waste. Cyclic voltammetry (CV) and electrochemical impedance spectroscopy (EIS) analysis confirmed that the material had rich and strong redox-active centers on its surface, indicating that it has potential to be used as a redox mediator for electron transfer. Fe_3_O_4_@hydrothermal carbon was added into the anaerobic sludge treatment system for the collaboration of dye decolorization. The results showed that azo dye decolorization efficiency reached the maximum value (98.3%) with the presence of Fe_3_O_4_@hydrothermal carbon, which was 16.6% higher than control reactor (without Fe_3_O_4_@hydrothermal carbon added). In addition, Fe_3_O_4_@hydrothermal carbon exhibits good reusability and the dye decolorization rates in the “anaerobic sludge–material” combining system were significantly higher than that in the “sludge-alone” system during the semi-continuous wastewater treatment process. Mechanistic investigations revealed that the enhanced decolorization is driven by a synergistically constructed interspecies electron transfer pathway. Specifically, the addition of Fe_3_O_4_@hydrothermal carbon improved the formation of the extracellular polymeric substance (EPS), which had positive effects on sludge stability and its interaction with the material. CV and electron transport system (ETS) activity analysis showed that the sludge exhibited high electrochemical activities with the support of the material, which led to a high electron transfer efficiency between the electron-donating and accepting microbial pairs in the treatment system. The high-throughput sequencing analysis showed that the structure of the microbial community changed during the semi-continuous treatment process; *Megasphaera* and *Clostridium* accounted for more than 87.5% of the total abundance of the bacterial community in the anaerobic sludge with material addition. Driven by the material-mediated process, these enriched functional taxa exhibited a high electron transfer efficiency between electron-donating and accepting pairs, accelerating the catalytic cleavage of azo bonds and ultimately improving the overall anaerobic treatment performance.

## Simple Summary

In this study, Fe_3_O_4_@hydrothermal carbon was successfully synthesized from rice straw waste. The material had a relatively high Brunauer–Emmett–Teller surface area, and cyclic voltammetry (CV) and electrochemical impedance spectroscopy (EIS) confirmed abundant redox-active centers on its surface, indicating its potential as a redox mediator. During semi-continuous treatment, dye decolorization in the “material–anaerobic sludge” system was significantly higher than in the “sludge-alone” system. The addition of Fe_3_O_4_@hydrothermal carbon improved the formation of extracellular polymeric substance (EPS), which had positive effects on sludge stability. CV and electron transport system (ETS) activity analysis showed that the material enhanced sludge electrochemical activity and electron transfer efficiency. High-throughput sequencing revealed that *Megasphaera* and *Clostridium* accounted for more than 87.5% of the bacterial community in sludge with material addition. Species from these two genera could directly catalyze azo bond cleavage and be involved in the direct interspecies electron transfer process mediated by the material, resulting in the improvement of the dye decolorization performance in the treatment system.

## 1. Introduction

Synthetic dyes have been widely applied in various areas of industries [1]. More than 100,000 kinds, with a total production of more than 1 million tons of dyes, were produced every year [2]. Among these dyes, the group of azo dyes (which are characterized by containing azo bonds inside their chemical structure) accounted for 70% of the total production of dyes [3]. Unfortunately, above 10% of azo dyes were discharged into the environment with wastewater without any treatment during the production and usage process [4]. Most azo dyes and their intermediate metabolites have serious negative effects on humans and animals, including carcinogenesis, teratogenesis, mutagenicity and so on [5]. Thus, wastewater containing azo dyes must undergo harmless treatment before it can be released into the environment.

At present, the most widely used biological method for azo dye wastewater treatment is the anaerobic sludge process, because oxygen in the aerobic environment usually competes with azo dyes for electrons which prevents the following azo dye reduction [5]. The diversity of microorganisms in the anaerobic sludge has complementary functions for the biodegradation of various azo dyes [6]. Compared with the aerobic treatment process, the anaerobic process can withstand higher organic loads, and is more cost-effective because it does not need energy for ventilation and agitation during the treatment process [7].

The main limiting factor affecting the large-scale application of anaerobic technology for azo wastewater treatment is the low treatment efficiency. The anaerobic process usually sustains for several days, which seriously delays the whole wastewater treatment progress [8]. Generally speaking, the first step for azo compound degradation is cleavage of the azo bonds by a reduction reaction catalyzed by various reductases [5]. Thus, the rate of electron transfer in the anaerobic system determines the azo dye reduction efficiency. On the one hand, the microorganisms inside the sludge need sufficient electron supply from their surrounding culturing environment. On the other hand, the electrons need to transfer from the microorganisms to dyes to cleave the azo bonds for dye reduction [9]. Thus, the higher rate of electron transfer between microorganisms and azo compounds and the higher efficiency of azo dye degradation can be attained in the anaerobic system. It has been widely reported that some chemicals, such as quinone compounds, can be used as redox mediators to help electron transfer between microorganisms and azo dyes. The quinones with simple chemical structures can enter the cells of microorganisms and be biocatalyzed to hydroquinones, and then the hydroquinones transfer to outside of the microbial cells and reduce the azo dyes to aniline compounds by a purely chemical redox reaction [10,11]. However, the anaerobic system usually lacks extra electron supply to microorganisms, resulting in the low efficiency of reductase to catalyze azo dye reduction. Thus, the enhancement of the extra electron supply level from the external environment to microorganisms in the anaerobic sludge may further increase azo dye degradation efficiency during the treatment process.

In recent years, a series of new carbon-based materials (such as activated carbon, biochar, hydrothermal carbon, carbon nanotubes and graphene) have caught the interest of researchers in the field of pollutant treatment [12,13,14]. First, the carbon-based materials can be prepared easily at low cost from various types of agricultural and industrial wastes, resulting in a large amount of waste being consumed at the same time as the preparation of functional materials [15,16]. Second, the prepared materials usually present as lamellar or spherical structure with loose and porous characteristics, which give them a large surface area for pollution adsorption [17]. Third, the prepared carbon-based materials usually have conductive properties, making them used as redox mediators in the reaction system for pollutant treatment [18,19]. Many studies have proved that the addition of carbon-based materials into the mixed biological treatment (including both bacterial consortia and sludge) can improve the efficiency of removing various pollutants [20].

Unfortunately, the practical application of carbon-based materials in wastewater treatment is often constrained by their difficult recovery from treated effluents. Once introduced into treatment systems, these materials may become mixed with sludge and subsequently disposed of together with residual solids, thereby creating potential secondary pollution. Magnetic modification of carbon materials provides a feasible solution to this challenge. In particular, carbon composites integrated with iron-based magnetic species can be readily separated using an external magnetic field and may exhibit enhanced reactivity toward pollutants. Moreover, magnetite also possesses conductive properties that can further facilitate microbial electron transfer. However, the potential of rice straw-derived magnetic hydrothermal carbon to accelerate anaerobic azo dye biodegradation has not been systematically evaluated, and the underlying electron transfer mechanisms remain unclear.

In this study, magnetic hydrothermal carbon was prepared using rice stalk waste as substrate. The adsorption and electrochemical activities of the material were tested to evaluate its potential for wastewater treatment. After that, the material was added into the anaerobic sludge system for synergistic treatment of azo dye wastewater. The acceleration performance of magnetic hydrothermal carbon on azo dye decolorization in the anaerobic treatment system was evaluated. The effects of the materials on the changes in sludge morphological characteristics, biological functions, electrochemical activities, and microbial community structure during the wastewater treatment process were determined. Our study will provide new insight into the resource utilization of agricultural waste at the same time as a technological process for the rapid treatment of azo dye wastewater.

## 2. Materials and Methods

### 2.1. Materials and Chemicals

In this study, straw of the rice cultivar Daohuaxiang No. 2 (collected from Wuchang county, Harbin, China), which is rich in cellulose, was adopted as the raw material for hydrothermal carbon synthesis. The anaerobic granular sludge (AGS) which was originally used for treatment of domestic effluent was obtained from Shuierli Environmental Protection Technology Co., Ltd. (Nanchang, Jiangxi, China). The azo dye, Chrome Black T, was selected as a model azo dye in this study, and was purchased from Sinopharm Chemical Reagent Co., Ltd. (Shenyang, China). The DNA extraction kit was procured from Omega Bio-tek (Norcross, GA, USA). All the other chemical agents were commercially available and of analytical grade.

### 2.2. Preparation and Characterization of the Magnetic Hydrothermal Carbon

Rice straw was thoroughly rinsed with deionized water to remove ash and surface impurities, dried at 60 °C for 24 h, and subsequently ground into powder. After sieving with a 200-mesh screen, 5 g of the fine powders was collected and added into 50 mL distilled water and shaken vigorously for thorough mixing. The reaction system was then transferred to an autoclave with the volume of 100 mL, and incubated in an oven at 180 °C for 8 h. The reaction product was removed from the autoclave until it cooled to room temperature and washed 3 times with deionized water. The product was collected and dried by vacuum filtration and lyophilization, respectively. The dried powders were used as hydrothermal carbon. Fe_3_O_4_@hydrothermal carbon was synthesized based on a conventional chemical co-precipitation method [18]. Briefly, 0.3 g of the prepared hydrothermal carbon particles was added into 150 mL of distilled water. Then, 0.70 g of FeCl_3_·6H_2_O and 0.72 g of FeSO_4_·7H_2_O were added into the distilled water, respectively. The reaction system was first stirred anaerobically at room temperature for 1 h. After adjusting the pH to 12, the reaction system was moved to a 60 °C water bath and incubated for another 2 h under anaerobic conditions. The final products could be separated from the solution by a strong magnet. The synthesized products were repeatedly washed with deionized water and absolute ethanol until neutral pH was achieved and subsequently freeze-dried under vacuum at −40 °C for 24 h.

The morphological characteristics of the prepared hydrothermal carbon and Fe_3_O_4_@hydrothermal carbon were observed by a scanning electron microscope (SEM) equipped with energy-dispersive spectroscopy (EDS) (TM3030, Hitachi, Tokyo, Japan) at a voltage of 15 kV. The functional groups on the surface of materials were measured via Fourier transform infrared spectroscopy (FT-IR) which was performed on a Thermo Fisher Scientific nicolet is50 spectrometer (Thermo Nicolet Corporation, Madison, WI, USA) in the frequency range of 500–4000 cm^−1^ at a resolution of 4 cm^−1^ by making the KBr thin pellet with the test sample. The measurement of Brunauer–Emmett–Teller (BET) surface area of the prepared materials was performed on a 3H-2000 automatic specific surface area analyzer with nitrogen adsorption/desorption at 77 k (Beishide Instrument Technology Co., Ltd., Beijing, China).

The CV tests on the materials prepared in this study were performed with the three-electrode electrochemical system on an electrochemical workstation (CHI 760E, Shanghai Chenhua Instrument Co., Ltd., Shanghai, China). Ten milligrams of each prepared material was uniformly coated onto a glassy carbon electrode with a 3 mm diameter and used as the working electrode. Prior to measurements, the glassy carbon electrode was polished with 0.05 μm alumina powder and ultrasonically cleaned in ethanol for 30 s. A platinum foil electrode was used as the counter electrode while a Ag/AgCl electrode was used as the reference electrode. The CV tests were performed at a voltage range of −1.4 V to 1.4 V with a scan rate of 20 mV/s in 0.01 mol/L PBS buffer (pH = 7.2). Electrochemical impedance spectroscopy (EIS) measurements were conducted in 0.01 mol/L PBS (pH 7.2) under open-circuit conditions, with an AC amplitude of 10 mV over a frequency range from 10 MHz to 100 KHz [21].

### 2.3. The Adsorption Tests of the Prepared Materials

The capacities of the prepared materials for dye adsorption were detected. In brief, 50 mg of hydrothermal carbon or Fe_3_O_4_@hydrothermal carbon was added into 50 mL PBS buffer (pH = 7.0) containing 50 mg/L Chrome Black T, respectively. The treatment systems were incubated for 9 h at 30 °C under dark conditions. After the adsorption process, the supernatant was collected by centrifugation (10,000× *g*, 5 min), and was subsequently analyzed on a UV spectrophotometer at the wavelength of 540 nm. The degradation kinetics of Chrome Black T were analyzed using the pseudo-first-order rate model, as expressed by the following equation:

ln(C0Ct)=kt

where *C*_0_ indicates the initial concentrations of the Chrome Black T solution (mg/L); *C_t_* refers to the concentration of Chrome Black T at time *t* (mg/L); the parameter *k* is the pseudo-first-order rate constant; and *t* represents the adsorption time (h) [22].

### 2.4. Anaerobic Reactor Construction and Sludge Acclimation

The anaerobic reactor used in this study was a 10 L of PVC vessel equipped with a stirrer for mixing the sludge and medium. The initial sludge was added into the anaerobic reactor with the ratio of volatile suspended sludge (VSS): total suspended sludge (TSS) = 0.7. During the operation process, the temperature was kept at 30 °C, and the oxygen concentration was kept below 0.3 mg/L. The compositions of the basal medium used for anaerobic reactor operation were as follows: 4 g/L glucose, 0.4 g/L NH_4_Cl, 0.25 g/L KH_2_PO_4_, 0.008 g/L MgCl_2_, 0.002 g/L CaCl_2_ and 1 mL/L trace element solution [23]. The initial pH of the basal medium was adjusted to 7.0.

The anaerobic reactor was operated at a hydraulic retention time (HRT) of 24 h with the filling phase of 30 min, and the reaction phase of 21.5 h, a settling phase of 1 h, a draw phase of 45 min, following an idle time of 15 min. At the beginning of the acclimation process, the treatment system was operated with the azo dye free basal medium for 30 days. After that, the medium containing 50 mg/L of Chrome Black T was added into the reactor every day; the reactor was operated under this condition for 15 days. Over the next 75 days, the dye concentration in the reactor was increased to 300 mg/L, gradually (increasing 50 mg/L of the azo dye for every fifteen days of operation). After the acclimation process, the anaerobic reactor was operated for another 30 days for stabilization.

### 2.5. Batch Assays

After the acclimation process, different combining systems were constructed to evaluate the synergistic effect of the carbon-based materials on the azo dye decolorization with the anaerobic sludge. Briefly, 80 mL of basic medium was filled into a 100 mL serum bottle and was flushed with N_2_ for 20 min to expel O_2_. After that, 1.10 g dry weight of the acclimated sludge was inoculated to the medium, and Chrome Black T was added into the bottle to make sure its final concentration was 300 mg/L. The bottles containing the above ingredients were divided into three groups. Four-hundred milligrams of hydrothermal carbon or Fe_3_O_4_@hydrothermal carbon was added into the first two bottles, respectively. The bottle without material addition was used as the control group. The different treatment systems were incubated under 30 °C for 9 h, and 1 mL of sample in each bottle was taken out at different intervals to determine the dye degradation rate and the COD removal rate in each bottle.

The decolorization rate of Chrome Black T was estimated by measuring the absorbance at 540 nm with an ultraviolet visible spectrophotometer (UV-2800, Unico Instrument Co., Ltd., Shanghai, China). COD was measured using rapid digestion spectrophotometry in a strong acid medium and detected at a wavelength of 600 nm [24].

After the dye decolorization process, the effluent in the “sludge–Fe_3_O_4_@hydrothermal carbon” system was collected and the intermediate products after Chrome Black T biodegradation were identified using an LC-MS system (Waters Corporation, Milford, CT, USA). All effluent samples were pre-treated by filtration with a 0.22 μm filter. Chromatographic separation was performed on a Waters XTerra C18 column (2.1 × 100 mm, 1.2 μm). The mobile phase consisted of ultrapure water containing 0.1% formic acid (Solvent A) and acetonitrile (Solvent B). Gradient elution was conducted as follows: Solvent B was held at 60% for 17 min, and then decreased to 10% over the subsequent 7 min. The flow rate was 0.4 mL/min, and the injection volume was 10 μL. Mass spectrometry was operated in positive electrospray ionization (ESI) mode with an m/z range of 90–800. The nitrogen gas flow rate was 12 L/min, drying gas temperature was 350 °C, nebulizer pressure was 70 psi, and capillary voltage was set to 8000 V.

### 2.6. Semi-Continuous Test

The sludge was inoculated into different anaerobic treatment systems (0.5%, *w*/*v*) containing 300 mg/L of Chrome Black T to evaluate the dye decolorization performance in the different systems within a relatively long time span. The treatment system containing sludge and Fe_3_O_4_@hydrothermal carbon was considered as the test group. The anaerobic system that only contained sludge was used as the control group. The systems were operated for 15 days at a HRT of 24 h which we mentioned above. The dye decolorization rate in different operation systems was measured during the whole treatment process.

All experiments were performed in at least three independent replicates. Data analysis and plotting were conducted using GraphPad Prism 10.1.2 and Origin 2021. A *t*-test was applied to evaluate the statistical significance between different treatment groups.

### 2.7. Extraction and Characteristic Analysis of Extracellular Polymeric Substances (EPS) of the Sludge

EPS was extracted from the anaerobic sludge before or after the semi-continuous treatment process in different groups with a thermal extraction approach [25]. Briefly, 20 mL of sludge in each group was collected and washed 3 times using 0.5% NaCl solution. Then, the sludge sample was re-suspended in 20 mL of the same solution, and the whole system was heated to 80 °C and maintained for 45 min. After the incubation process, the supernatant was collected by centrifugation (10,000× *g*, 20 min) and considered as the crude EPS.

The contents of polysaccharide (PS) and protein (PN) in EPS were examined by a UV/VIS spectrophotometer (UV-2800, Unico Instrument Co., Ltd., Shanghai, China) according to the anthrone method with glucose as the standard, and the Lowry–Folin method with bovine serum albumin as the standard, respectively [26]. EPS was electrochemically characterized by CV at a voltage range of −0.6 V to 0.6 V with a scan rate of 0.1 V/s. The production of cytochrome C in EPS was tested using a UV/VIS spectrophotometer with absorbance scanning from 300 to 600 nm, in 1 nm increments.

### 2.8. Morphological and Electrochemical Characteristics of the Anaerobic Sludge in Different Systems After the Semi-Continuous Treatment

After the semi-continuous treatment process, the sludge and the effluent in different groups were separated by centrifugation (8000× *g*, 5 min). The morphological characteristics of the sludge after the treatment process were observed by a SEM.

Electrochemical properties of the sludge and the effluent were characterized using an electrochemical workstation (CHI 760E, Shanghai Chenhua Instrument Co., Ltd., Shanghai, China). For the CV test of the effluent before or after the treatment, a graphite plate, a platinum plate and a Ag/AgCl electrode were used as the working, calculation and reference electrodes, respectively. The CV test was performed in the range of −1.0 V to 1.2 V at a scan rate of 100 mV/S. After washing with 0.01 mol/L PBS buffer (pH = 7.0) 3 times, the anaerobic sludge was immobilized on two parallel gold electrodes with a 0.5 mm insulation gap. A voltage ramp of −0.3 V to 0.3 V with a step increment of 0.025 V was applied to determine the conductivity of the sludge [27].

### 2.9. High-Throughput Sequencing Analysis

The bacterial community structure of the anaerobic sludge with hydrothermal carbon or Fe_3_O_4_@hydrothermal carbon addition before or after the semi-continuous treatment was determined using Illumina high-throughput sequencing. The total DNA of the sludge in the different treatment systems was extracted using a DNA extraction kit according to the manufacturer’s instructions. The quality and concentration of the DNA sample were examined by a NanoDrop ND-2000 spectrophotometer (NanoDrop Technologies Inc., Wilmington, DE, USA). The primers 515F (5′-GTGCCAGCMGCCGCGGTAA-3′) and 806R (5′-GGACTACHVGGGTWTCTAAT-3′) were used for bacterial partial 16s RNA gene amplification. After purification, the PCR products were sequenced on an Illumina Nova 6000 PE250 platform (Illumina, San Diego, CA, USA) according to the standard protocols by Magigene Biotechnology Co., Ltd. Company (Guangzhou, China). High-quality reads were clustered into operational taxonomic units (OTUs) by the uparse algorithm with 97% identity [28]. The taxonomy of OTUs was determined based on the SILVA 132 database, and the confidence threshold was set to the default of ≥0.8 [29].

## 3. Results and Discussion

### 3.1. Characterization of the Prepared Materials

**Figure 1:**
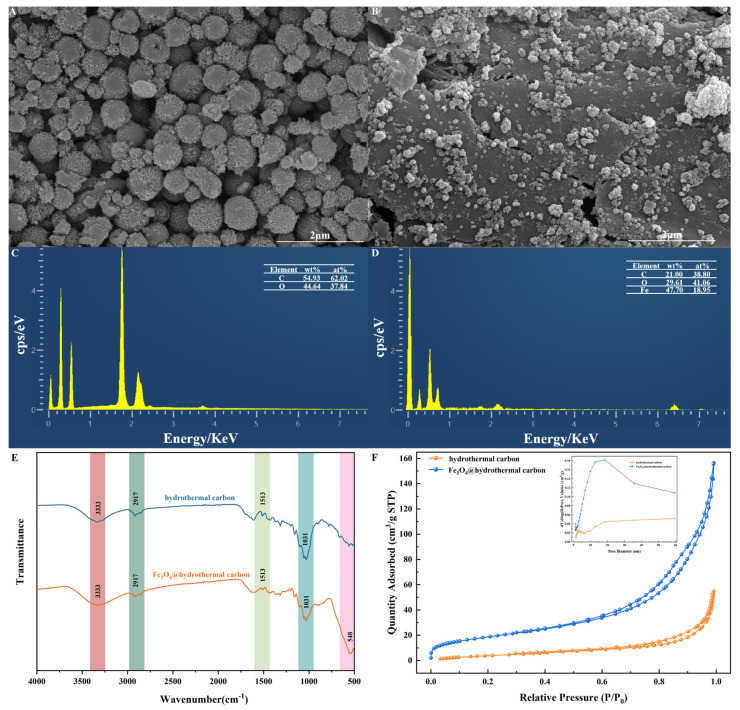
Characterization of hydrothermal carbon and Fe_3_O_4_@hydrothermal carbon. SEM images of (**A**) hydrothermal carbon and (**B**) Fe_3_O_4_@hydrothermal carbon; EDS spectra of (**C**) hydrothermal carbon and (**D**) Fe_3_O_4_@hydrothermal carbon; (**E**) FTIR spectra of the two prepared materials; (**F**) adsorption and desorption curves of N_2_ adsorption for the prepared materials; the pore size distributions of the materials are shown on the right in the panel.

SEM analysis was used to evaluate the morphological characteristics of the two prepared materials. As shown in Figure 1A,B, both of the materials presented lamellar structures. Moreover, many honeycomb holes could be found on the surface of the two materials, which might be due to the removal of volatile substances in the rice straw during the pyrolysis process. Compared with hydrothermal carbon, some granules with diameters from 10 to 20 nm exhibiting inverse spinel structures could be observed on the surface of the improved material, indicating that Fe_3_O_4_ was synthesized successfully and adhered on the surface of hydrothermal carbon [18]. EDS analysis showed that about 62% of the element in hydrothermal carbon was C. Besides C element, strong signals of Fe and O could be detected in the Fe_3_O_4_@hydrothermal carbon, and the ratio of the three elements was close to 2:2:1, which further confirmed that Fe_3_O_4_ had already bound to the surface of hydrothermal carbon (Figure 1C,D). Fe_3_O_4_ gave the modified material its magnetic property, which might help the separation of the material and wastewater after the treatment process. Moreover, with the rough and fluffy surface, the modified material made it easier for microorganisms to attach to its surface [21].

The FTIR spectra of the two prepared materials are shown in Figure 1E. A series of functional groups presented on the surface of the two materials. For example, the two peaks at 3333 cm^−1^ and 2917 cm^−1^ indicated the presence of –OH and C–H groups on the surface of the materials; the peak at 1513 cm^−1^ and 1031 cm^−1^ represented the C=O and C–O stretching vibrations of quinonyl and alkoxy [30]. It is worth mentioning that the signals of most of functional groups in the spectrum of Fe_3_O_4_@hydrothermal carbon were higher than in those of the spectrum of hydrothermal carbon, indicating that Fe_3_O_4_@hydrothermal carbon might more likely be involved in an oxidation-reduction catalytic process during the pollution treatment process. Compared with hydrothermal carbon, a unique broad peak at 548 cm^−1^, which corresponded to Fe-O stretching vibrations, could be found in the spectrum of Fe_3_O_4_@hydrothermal carbon, indicating that iron oxides were attached on the surface of hydrothermal carbon [22].

The N_2_ adsorption–desorption isotherms of both of the two materials displayed a type IV curve and H3 hysteresis loop according to the International Union of Pure and Applied Chemistry (IUPAC) classification, which showed that both hydrothermal carbon and Fe_3_O_4_@hydrothermal carbon exhibited typical mesoporous structures, giving the two materials a relative big surface area for dye adsorption and concentration. The BET surfaces of hydrothermal carbon and Fe_3_O_4_@hydrothermal carbon were 18.3 and 69.6 m^2^/g, respectively. Meanwhile, a more concentrated pore size distribution could be observed on the surface of the modified material (Figure 1F). The results indicated that introducing Fe_3_O_4_ to the hydrothermal carbon increased the surface area of the material as well as improved the homogeneity of the material structure [31].

**Figure 2:**
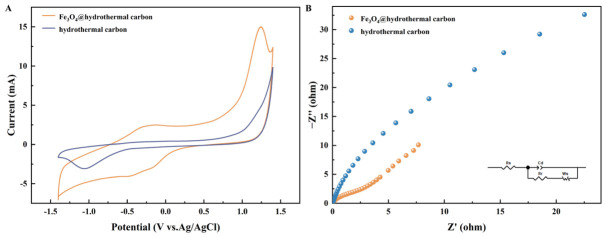
Electrochemical analysis of hydrothermal carbon and Fe_3_O_4_@hydrothermal carbon materials. (**A**) Cyclic voltammogram of the two materials; (**B**) EIS analysis of the two materials.

The electrochemical CV was conducted to evaluate the redox properties of the two materials prepared in this study (Figure 2A). It could be observed that both hydrothermal carbon and Fe_3_O_4_@hydrothermal carbon exhibited conductive properties. Compared with hydrothermal carbon, Fe_3_O_4_@hydrothermal carbon exhibited redox peaks, which suggested that Fe_3_O_4_@hydrothermal carbon had richer and stronger redox-active centers on its surface. Moreover, the integral area of the cyclic voltammetry closed curve of Fe_3_O_4_@hydrothermal carbon was larger than that of the hydrothermal carbon, indicating the modified material had a higher capacitance than the traditional material, which gave the material higher electroactivity to store and release extra electrons [32].

EIS analysis was carried out to further investigate the electron transfer capability of the two materials. As shown in Figure 2B, Fe_3_O_4_@hydrothermal carbon possessed a smaller circular arc in the high-frequency region and a larger slope in the low-frequency region than those of the hydrothermal carbon. The results indicated that Fe_3_O_4_@hydrothermal carbon had lower ohmic internal resistance and internal charge transfer resistance, which gave it higher charge transfer efficiency [33]. The higher electrochemical activities of exhibited by Fe_3_O_4_@hydrothermal carbon might be attributed to the doping of Fe_3_O_4_ into hydrothermal carbon; the Fe ions in the material could accept or release electrons efficiently by changing their valence state, resulting in enhanced conductivity of the material [30]. All the results mentioned above indicated that Fe_3_O_4_@hydrothermal carbon could be used as an ideal redox mediator for electron transfer between the microbial cells in the anaerobic sludge.

### 3.2. The Adsorption Performance of the Two Prepared Materials

**Figure 3:**
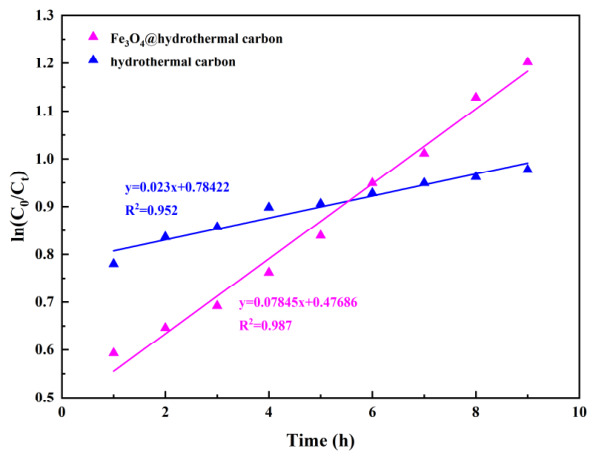
Pseudo-first-order kinetic model for the adsorption of azo dye onto hydrothermal carbon and Fe_3_O_4_@hydrothermal carbon.

It could be observed that the adsorption of Chrome Black T by both the hydrothermal carbon and Fe_3_O_4_@hydrothermal carbon followed the pseudo-first-order kinetic model, and the dye decolorization rate could be up to 70% in the two systems after a 9 h adsorption process (Figure 3). It is interesting to see that, in the first half of the adsorption process, hydrothermal carbon exhibited a better adsorption performance than Fe_3_O_4_@hydrothermal carbon. It might be attributed to the agglomeration of Fe_3_O_4_ on the surface of hydrothermal carbon, which reduced the accessibility of active sites on the surface of the improved material [22]. However, in the following 4 h incubation process, the adsorption efficiency of the improved material was higher than that of the traditional material, which might be attributed to the double-walled structure of Fe_3_O_4_@hydrothermal carbon. After the initial adsorption of Chrome Black T on the outer surface of the improved material, the dye molecules diffused into the porous inner layer via size-selective pathways, which gave the material more capacity for dye adsorption [34].

### 3.3. Batch Assays of Azo Dye Decolorization in Different Treatment Systems

**Figure 4:**
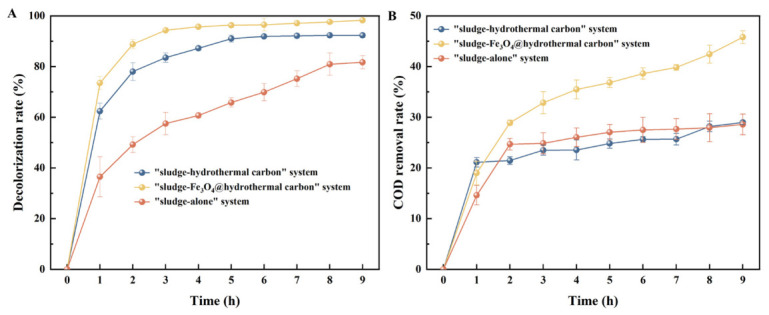
The azo dye treatment performances of the different treatment systems in the batch assays. (**A**) The dye decolorization rate; (**B**) the COD removal rate.

The azo dye decolorization performances in different treatment systems were tested (Figure 4A). The results showed that the addition of carbon-based materials had a synergistic effect with the anaerobic sludge on dye decolorization. It could be observed that the dye decolorization rate only obtained around 80% after a 9 h treatment in the “sludge-alone” system. However, more than 92% of the decolorization rate could be obtained in the system with hydrothermal carbon addition after the treatment process. The highest azo dye decolorization efficiency was obtained in the “sludge–Fe_3_O_4_@hydrothermal carbon” combining system; more than 94% of azo dye was already decolorized within 3 h in this system. It is worth mentioning that although the dye decolorization efficiency in the “sludge–hydrothermal carbon” system was higher than that of in the “sludge-alone” system, no significant enhancement of the COD removal rate could be obtained. The final COD removal rates of the two systems were all around 30%, indicating that a large amount of intermediates were acclimated in the anaerobic system after the dye decolorization, which could not be further biodegraded by the sludge (Figure 4B). Interestingly, the COD removal rate in the “sludge–Fe_3_O_4_@hydrothermal carbon” combining system was much higher than that of the other two systems, indicating that the interaction between different types of microorganisms was improved by the addition of Fe_3_O_4_@hydrothermal carbon; parts of the intermediates produced after the dye decolorization could be further biomineralized depending on the synergistic effects of the different microorganisms [20]. Considering Fe_3_O_4_@hydrothermal carbon had better electrochemical activities and better synergistic dye decolorization effects with the anaerobic sludge, we selected it for our further study.

LC-MS analysis was performed to identify the intermediates generated after dye decolorization in the “sludge–Fe_3_O_4_@hydrothermal carbon” system. One of the degradation products resulting from the cleavage of the azo bond in Chrome Black T, namely 4-amino-3-hydroxy-7-nitronaphthalene-1-sulfonic acid, was successfully detected in the treated wastewater (Figure S1). This result indicates that the anaerobic treatment system effectively disrupted the azo bond structure of the dye and transformed the parent compound into aromatic amine-related intermediates, thereby achieving decolorization. However, although efficient dye decolorization was observed, these intermediates could not be completely mineralized under anaerobic conditions and therefore remained in the treated effluent. The persistence of these degradation intermediates is likely responsible for the incomplete COD removal observed in this study.

### 3.4. Decolorization of Chrome Black T by the Different Anaerobic Treatment Systems Under Semi-Continuous Conditions

**Figure 5:**
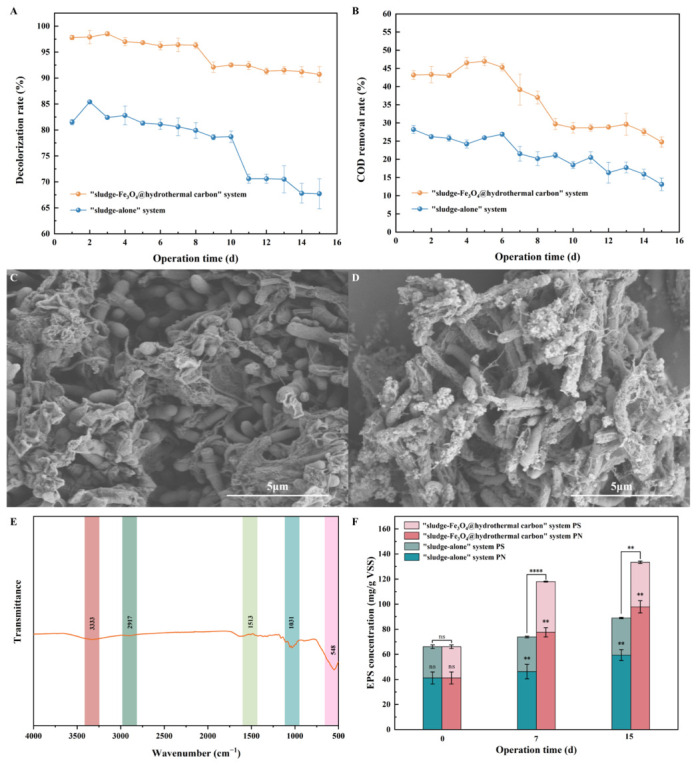
Decolorization of Chrome Black T in the different treatment systems under semi-continuous conditions. (**A**) The dye decolorization rate in the different systems during the whole treatment process; (**B**) the COD removal rate in the different systems during the whole treatment process; the SEM image of the sludge after the treatment process in the (**C**) “sludge-alone” system and (**D**) the “sludge–Fe_3_O_4_@hydrothermal carbon” system; (**E**) FTIR spectra of Fe_3_O_4_@hydrothermal carbon after semi-continuous treatment; (**F**) concentrations of PN and PS in the EPS of the sludge in the different treatment systems. Each experiment was repeated three times: ns > 0.05, ** *p* ≤ 0.01, **** *p* ≤ 0.0001.

In this study, the anaerobic sludge with or without Fe_3_O_4_@hydrothermal carbon addition was used for the semi-continuous wastewater treatment tests; the daily decolorization rates of the azo dye in the different systems were detected during the whole operation process (Figure 5A). It could be observed that both of the systems with or without the prepared material could decolorize the azo dye effectively during the first five operation cycles. However, the dye decolorization efficiency decreased gradually in the sludge-alone system from the sixth treatment cycle; only 78% of dye could be decolorized in the tenth treatment cycle; and finally, the decolorization decreased to 67% in the last cycle of the operation process. Meanwhile, the COD removal efficiency in the “sludge-alone” system gradually decreased during the semi-continuous operation process. On day 15, the COD removal rate dropped to only 13.1% (Figure 5B). The SEM image shows that partial bacterial cells in the sludge became deformed and even ruptured after continuous exposure to high concentrations of dye (Figure 5C). The results indicated that the sludge could not sustain the long-term force of the wastewater. The toxic effects of dyes on microorganisms significantly decreased the biological activities of the anaerobic sludge. In comparison, the “sludge–Fe_3_O_4_@hydrothermal carbon” system could maintain its high efficiency on dye decolorization. The decolorization rate of Chrome Black T remained above 90% in every cycle during the whole treatment process. The SEM image showed that the microorganisms in the sludge maintained their shape and structure after the whole treatment process (Figure 5D).

To further address the structural stability of Fe_3_O_4_@hydrothermal carbon during the decolorization process, FTIR analysis was performed for the material after the semi-continuous process (Figure 5E). It could be observed that the major characteristic functional groups of the material (including –OH, C–H, C=O, C–O and Fe–O) were largely retained after the semi-continuous treatment, indicating that the material maintained its fundamental chemical structure during the operation process.

Moreover, it could be observed that the addition of Fe_3_O_4_@hydrothermal carbon led to a significant increase in PN and PS contents in the EPS of the anaerobic sludge with material addition (Figure 5F). After the 15 days of operation, the concentrations of PN and PS in the test group were 2.38- and 1.43-times higher than of those in the primary sludge. The high efficiency of sludge on PN and PS secretion might due to the favorable micro-environment created by Fe_3_O_4_@hydrothermal carbon for the microorganisms in the sludge. The higher contents of PN and PS, in turn, further improved the adhesion of microorganisms on the surface of the prepared material which shortened the electron transfer distance between Fe_3_O_4_@hydrothermal carbon and microorganisms, thus increasing the efficiency of pollutant degradation [32]. Moreover, the adhesion effects of PN and PS also led to the granulation of the sludge to maintain its stability during the wastewater treatment process [35]. As shown in Figure S2A, EPS extracted from the “sludge–Fe_3_O_4_@hydrothermal carbon” system exhibited a substantially higher oxidation peak current than that from the “sludge-alone” system after 7 days of semi-continuous operation. The increased oxidation peak current indicates enhanced electron-donating capacity (EDC) of EPS. Previous studies have shown that conductive carbonaceous materials and iron-containing particles can stimulate microorganisms to produce greater amounts of redox-active compounds, including humic-like substances and cytochrome proteins, within the EPS matrix. These compounds function as electron shuttles and facilitate extracellular electron transfer. Consequently, the enhanced redox activity of EPS directly supports the accelerated dye decolorization observed in our study.

Moreover, as shown in Figure S2B, cytochrome c production within EPS increased by 85.1% in the “sludge–Fe_3_O_4_@hydrothermal carbon” system, compared with only 12.5% in the “sludge-alone” system. These results suggest that Fe_3_O_4_@hydrothermal carbon promoted the accumulation of redox-active proteins involved in electron transport. Because cytochrome c is an essential electron transfer component in anaerobic respiration and may participate in DIET processes, its increased abundance further supports enhanced extracellular electron transfer.

### 3.5. Effect of Fe_3_O_4_@hydrothermal Carbon on the Electroactivity of the Anaerobic Sludge

**Figure 6:**
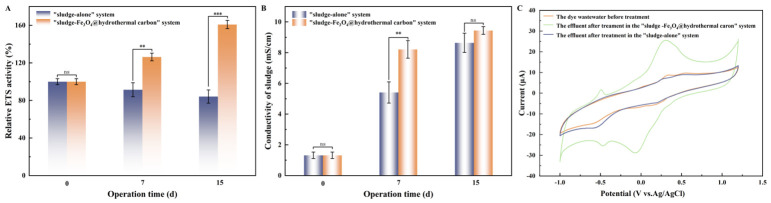
The electrochemical properties of the anaerobic sludge after the semi-continuous treatment in the different systems. (**A**) The ETS activities of the sludge; (**B**) the conductivity of the sludge; (**C**) CV curves of the effluent in the different treatment systems. Each experiment was repeated three times: ns > 0.05, ** *p* ≤ 0.01, *** *p* ≤ 0.001.

In our study, the ETS activity was assayed to evaluate the electron transfer activity of the anaerobic sludge. As shown in Figure 6A, in the “sludge-alone” system, the ETS activities of the sludge decreased gradually with the increase in the wastewater treatment cycle. Compared with the original sludge, only 91% and 84% of the relative activity of ETS could be retained after the 7- and 15-day semi-continuous process, respectively, indicating that the azo dye with high concentration had toxic effects on the sludge, which decreased its biological activities for electrons transfer. Interestingly, when the treatment system contained Fe_3_O_4_@hydrothermal carbon, the situation changed. It could be observed that the ETS activity of the sludge increased by 61% after the 15 days of operation, indicating that Fe_3_O_4_@hydrothermal carbon had a positive effect on maintaining the stability and the electrochemical activity of the anaerobic sludge. The conductivity of sludge in the test group was higher than that of in the control group during the whole semi-continuous treatment process (Figure 6B). The high conductivity of the sludge facilitated electron transfer between the different microorganisms in the sludge [36]. To further confirm this hypothesis, the extracellular electron transfer capacities of the different treatment systems were further evaluated by CV test. As shown in Figure 6C, no significant redox peaks could be observed in the effluent of the control group before or after the treatment of azo dye wastewater, indicating that few electrons were transferred between the different types of microorganisms in the treatment system. Comparatively, four distinct redox peaks at 0.302 V (vs. Ag/AgCl, oxidation peak), −0.491 V (vs. Ag/AgCl, oxidation peak), −0.471 V (vs. Ag/AgCl, reduction peak) and −0.066 V (vs. Ag/AgCl, reduction peak) could be detected in the effluent of the material–sludge combining treatment system.

The results mentioned above clearly showed that, in the “sludge–Fe_3_O_4_@hydrothermal carbon” system, the material could be used as an intermediate to accelerate the electron transfer efficiency between the electron donor microorganisms to azo dye degradation microorganisms to improve their relative key metabolic activities for azo dye reduction.

### 3.6. Microbial Community Structure Analysis

The bacterial community changes in the anaerobic sludge with or without Fe_3_O_4_@hydrothermal carbon addition after the semi-continuous wastewater treatment process were assessed using Illumina high-throughput sequencing analysis. The sequence information for the test samples is summarized in Table 1. After quality filtering, 322,039 clean reads were obtained in total for the three test samples. The OTUs of each sample ranged from 887 to 936. A high Good’s coverage was achieved for all samples. The Chao 1 richness index and Shannon diversity index showed that the diversity of the bacteria decreased after the semi-continuous treatment process in both the systems with or without material addition, indicating that some species of bacteria could not afford the impact of the azo dye with high concentration over a long treatment period, and they faded out from the sludge community gradually.

**Figure 7:**
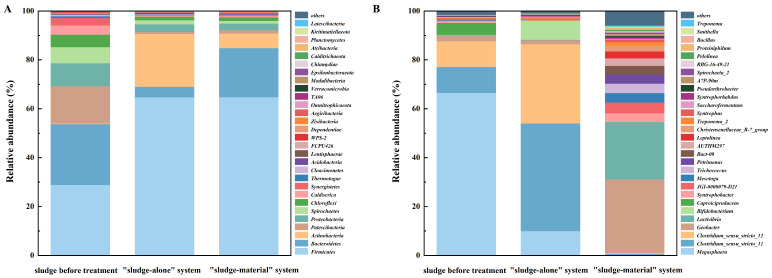
The bacterial diversity of the anaerobic sludge in the different treatment systems after the semi-continuous treatment process. (**A**) The phylum level; (**B**) the genus level.

The phylogenetic classification of the effective sequences is shown in Figure 7. At the phylum level, before the semi-continuous treatment, the main phyla in the sludge belong to *Firmicutes*, *Bacteroidetes*, *Actinobacteria*, *Patescibacteria Proteobacteria*, *Spirochaetes* and *Chloroflexi*. However, after the 15 days of operation, the phylum distribution of the microbial community changed significantly. Compared with the original sludge, the abundance of *Bacteroidetes*, *Patescibacteria*, *Proteobacteria* and *Spirochaetes* decreased, indicating that some of the microbial species belonging to these phyla could not adapt to the harsh environment containing a high concentration of azo dye, and it seemed like they did not play a crucial roles in dye decolorization. At the same time, *Firmicutes* became the dominate phylum in both of the “sludge-alone” and “sludge–Fe_3_O_4_@hydrothermal carbon” system after the treatment process, with the abundance increasing from 28.86% in the sample before treatment to 64.74% and 64.79% in the above two systems, respectively. In addition, the phylum *Actinobacteria* was also increased to some extent in the two treatment systems.

At the genus level, the dominant genera in the anaerobic sludge of the test group with material addition after the semi-continuous treatments were *Megasphaera*, *Clostridium*, *Geobacter*, and *Caproiciproducens*. Compared with the initial anaerobic sludge, the abundance of *Megasphaera* and *Clostridium* increased by 66.17% and 20.4%, respectively. The abundance of the two above genera accounted for more than 87.5% of the total abundance of the bacterial community in the anaerobic sludge. It is worth mentioning that both of the genera mentioned above were phylogenetic related, which belong to the *Clostridia* class [37]. Both the genera had high abilities for degradation of the substrates with complex chemical structures, and could be used for the bioremediation of various types of pollution. They were the predominant genera involved in the azo dye anaerobic treatment process in a series of anaerobic dying wastewater treatment systems, and were responsible for azo dye biodegradation [38]. On the one hand, they could degrade azo dyes directly. *Clostridium* was thought to be the predominant bacterial genus with the anaerobic azoreductase activity which catalyzed the cleavage of the azo bond [39]. *Megasphaera* also showed significantly positive correlations with azo bond cleavage under anaerobic conditions [40]. On the other hand, the bacterial species in *Clostridium* and *Megasphaera* genera could produce hydrogen after co-substrate fermentation, which could be used as a suitable electron donor for azo dye reduction by other bacteria in the anaerobic sludge [41]. Moreover, both *Clostridium* and *Megasphaera* could generate extra electrons during the carbohydrate oxidation process, and the species in the two genera were considered to be the electron-donating bacteria in the mixed microbial populations [42]. These species were inferred to have the ability of direct interspecies electron transfer to other azo dye degradation microorganisms. Some studies pointed out that the flagella structure of the *Clostridium* sp. might participate in the DIET process [43,44,45]. However, Wang et al. reported that the sludge conductivity remained at a low level during the wastewater treatment process in the pure bioreactor, indicating that owing conductive flagella of *Clostridium* sp. was not the main reason for the DIET establishment in the anaerobic system [46]. Therefore, conductive materials should be added to the treatment system to be used as the mediators for the DIET process. A series of carbon-based materials, such as biochar and carbon cloth, had been proved to have the ability to improve the DIET process between the electron-donating and electron-accepting microbial partners, resulting in the acceleration of different types of reduction reaction, including pollutant degradation and CH_4_ generation [47,48]. In our study, the prepared hydrothermal carbon exhibited conductive properties. Moreover, as a conductive iron material, Fe_3_O_4_ was also believed to have the ability to improve the DIET process [49]. Thus, the combination of hydrothermal carbon and Fe_3_O_4_ gave the Fe_3_O_4_@hydrothermal carbon higher electron conduction efficiency, resulting in a faster electron transfer rate between the electron-donating and electron-accepting bacteria in the anaerobic treatment system.

It was interesting to see that the abundance of the genus *Geobacter* decreased dramatically in both of the test and control systems after the semi-continuous wastewater treatment process. It is worth mentioning that *Geobacter* was one of the most important genera for mediating the DIET process in the anaerobic system [50]. It has been widely reported that the extracellular electron exchanges between *Geobacter* sp. and other bacteria via electrically conductive pili (e-pili) and cytochromes [51]. *Geobacter* sp. and other functional bacteria/archaea connect electrically through e-pili for more rapid electron transfer to promote a series of biochemical reactions (including CH_4_ production, biomass synthesis, and pollutant degradation) [52]. In the primary sludge, the abundance of *Geobacter* attained to 33%, indicated that the species belonging to *Geobacter* were responsible for the DIET process between the bacteria in the sludge without conductive material addition. This might be one of the reasons that the primary sludge had a good performance on dye decolorization during the acclimation process. However, the abundance decreased to 1.5% after the semi-continuous treatment process in the control group without material addition, indicating that the DIET mediated by *Geobacter* was no longer common in the anaerobic system, resulting in a low efficiency of dye reduction. During semi-continuous operation, the sludge was continuously exposed to high-concentration azo dye and glucose-rich influent. Under this condition, fast-growing fermentative bacteria, such as *Megasphaera* and *Clostridium*, could gain a competitive advantage because they efficiently utilize organic substrates and generate reducing equivalents, hydrogen, or other electron donors for azo dye reduction. In contrast, *Geobacter* is not generally regarded as a dominant primary glucose-fermenting microorganism, which may explain its gradual decline during semi-continuous operation. Fortunately, the decrease in *Geobacter* did not negatively affect the performance of the “sludge–Fe_3_O_4_@hydrothermal carbon” system. The conductive material likely served as an abiotic electron transfer conduit, partially compensating for the reduced biological electron transfer mediated by *Geobacter*. This interpretation is consistent with the enhanced sludge conductivity, increased ETS activity, stronger EPS redox activity, and sustained decolorization performance observed in the “sludge–Fe_3_O_4_@hydrothermal carbon” system.

In this study, Fe_3_O_4_@hydrothermal carbon enhanced the dye decolorization performance of anaerobic sludge through various mechanisms. First, the lamellar porous structure, large specific surface area, and abundant functional groups of Fe_3_O_4_@hydrothermal carbon facilitated the enrichment of dye molecules near the sludge–material interface while simultaneously providing favorable sites for microbial attachment and colonization. Second, the synergistic combination of the Fe_3_O_4_ phase and the carbon matrix endowed the material with enhanced redox activity and reduced charge transfer resistance. As a result, Fe_3_O_4_@hydrothermal carbon functioned as an effective electron transfer mediator within the anaerobic sludge system. Third, the addition of Fe_3_O_4_@hydrothermal carbon to the treatment system promoted the formation of a more electroactive and structurally stable EPS matrix of the sludge. Such an EPS network likely facilitated electron exchange among microorganisms, conductive materials, and azo dye molecules. Fourth, microbial community analysis revealed that Fe_3_O_4_@hydrothermal carbon significantly changed the bacterial community and enriched fermentative genera, such as *Megasphaera* and *Clostridium*. These microorganisms can efficiently metabolize organic substrates and produce reducing equivalents, hydrogen, and other electron donors required for azo dye reduction. Therefore, their enrichment likely increased electron availability within the anaerobic sludge system. Combined with the enhanced conductivity provided by Fe_3_O_4_@hydrothermal carbon, these electrons could be more efficiently transferred among microbial populations and ultimately utilized for azo bond reduction.

## 4. Conclusions

In conclusion, Fe_3_O_4_@hydrothermal carbon with good adsorption properties and active electrochemical activities was prepared using rice straw waste. The prepared material had synergistic effects with the anaerobic sludge on the treatment of dying wastewater. With the support of the material, the anaerobic sludge could maintain a high dye degradation efficiency during the whole semi-continuous treatment process. On the one hand, the sludge adsorbed tightly on the surface of the prepared material, which stabilized the structure of the anaerobic sludge as well as shortened the physical distance between the sludge and azo dye. On the other hand, the material improved the electrochemical activities of the sludge, resulting in a higher rate of electron transfer between the electron-donating and electron-accepting microorganisms in the treatment system. Under the influence of the added material, the structure of the bacterial community changed significantly. The two genera, *Clostridium* and *Megasphaera*, that both have the capacities for direct azo dye degradation and extracellular electron transfer dominated the microbial community in the material addition system. The species of the two genera cleave the azo bonds through the biocatalytic process as well as transfer extra electrons generated by organic compound fermentation to other dye degradation microorganisms using the material as a mediator.

Despite the promising decolorization performance, several challenges must be addressed before practical implementation can be realized. First, the COD removal efficiency remained substantially lower compared to the dye decolorization efficiency, indicating that the system primarily promoted azo bond cleavage and partial anaerobic transformation rather than complete mineralization of aromatic intermediates. Consequently, coupling the proposed process with subsequent aerobic treatment or advanced oxidation technologies may be necessary to achieve complete pollutant removal in industrial applications. Second, the enhanced DIET-mediated decolorization performance relied heavily on the availability of readily biodegradable carbon sources (e.g., glucose) as electron donors, which may be a limitation in real textile wastewater. Third, although rice straw serves as an abundant and low-cost carbon precursor, the overall economic viability of Fe_3_O_4_@hydrothermal carbon remains to be comprehensively evaluated. Finally, while the magnetic properties of Fe_3_O_4_@hydrothermal carbon facilitate rapid separation and recovery in laboratory-scale batch experiments, the efficient recovery, regeneration, and reuse of powdered hydrochar in large-scale continuous-flow anaerobic reactors remain significant engineering challenges.

## Figures and Tables

**Table 1:** Alpha-diversity of the bacterial community.

Samples	Reads	Shannon_2	Chao1	OTU	Good’s Coverage
“sludge–material” system	104,093	4.49	887.97	887	1
“sludge-alone” system	109,216	4.51	889.30	889	1
sludge before treatment	108,730	4.61	936.47	936	1

## Data Availability

Data will be made available on request.

## Supplementary Materials

The following supporting information can be downloaded at https://www.mdpi.com/article/10.3390/biology15120896/s1, Figure S1: Identification of metabolite of Chrome Black T by LC-MS analysis; Figure S2: (A) Electrochemical analysis of EPS; (B) The relative production of cytochrome C within EPS in the different treatment systems.

## References

[1] Cui D., Zhang H., He R., Zhao M. (2016). The comparative study on the rapid decolorization of azo, anthraquinone and triphenylmethane dyes by anaerobic sludge. Int. J. Environ. Res. Public Health.

[2] El Harfi S., El Harfi A. (2017). Classifications, properties and applications of textile dyes: A review. Appl. J. Environ. Eng. Sci..

[3] Sun L., Mo Y., Zhang L. (2022). A mini review on bio-electrochemical systems for the treatment of azo dye wastewater: State-of-the-art and future prospects. Chemosphere.

[4] Dutta S., Adhikary S., Bhattacharya S., Roy D., Chatterjee S., Chakraborty A., Banerjee D., Ganguly A., Nanda S., Rajak P. (2024). Contamination of textile dyes in aquatic environment: Adverse impacts on aquatic ecosystem and human health, and its management using bioremediation. J. Environ. Manag..

[5] Phan K.H., Le L.T., Ninh T.T.N., Tran C.S., Nguyen T.T., Nguyen D.T.D., Tra V.T., Tran T.D., Nguyen T.B., Mai T.P. (2024). Decolorization and degradation of azo dyes in thermophilic biological wastewater treatment process: A mini-review. Case Stud. Chem. Environ. Eng..

[6] Saratale R.G., Saratale G.D., Chang J.S., Govindwar S.P. (2011). Bacterial decolorization and degradation of azo dyes: A review. J. Taiwan Inst. Chem. Eng..

[7] Mishra S., Singh V., Ormeci B., Hussain A., Cheng L., Venkiteshwaran K. (2023). Anaerobic–aerobic treatment of wastewater and leachate: A review of process integration, system design, performance and associated energy revenue. J. Environ. Manag..

[8] Cui M.H., Sangeetha T., Gao L., Wang A.J. (2019). Efficient azo dye wastewater treatment in a hybrid anaerobic reactor with a built-in integrated bioelectrochemical system and an aerobic biofilm reactor: Evaluation of the combined forms and reflux ratio. Bioresour. Technol..

[9] Martinez C.M., Zhu X., Logan B.E. (2017). AQDS immobilized solid-phase redox mediators and their role during bioelectricity generation and RR2 decolorization in air-cathode single-chamber microbial fuel cells. Bioelectrochemistry.

[10] Guo J., Lian J., Guo Y., Liu X., Zhang C., Yue L., Wang Y. (2015). Redox activity and accelerating capacity of model redox mediators during biodenitrification. Biotechnol. Biotechnol. Equip..

[11] Shen D., Leng D., Li C., Wu C., Cui D. (2016). Effect of quinoid mediators on the decolorization of azo dyes by the strain CD-2. Adv. Chem. Eng. Sci..

[12] Wang Q., Zhang Y., Zheng Y., Fagbohun E.O., Cui Y. (2024). Magnetic activated carbon for the removal of methyl orange from water via adsorption and Fenton-like degradation. Particuology.

[13] Madima N., Mishra S.B., Inamuddin I., Mishra A.K. (2020). Carbon-based nanomaterials for remediation of organic and inorganic pollutants from wastewater. A review. Environ. Chem. Lett..

[14] Zhang J., Zhao W., Zhang H., Wang Z., Fan C., Zang L. (2018). Recent achievements in enhancing anaerobic digestion with carbon-based functional materials. Bioresour. Technol..

[15] Foroutan R., Peighambardoust S.J., Peighambardoust S.H., Pateiro M., Lorenzo J.M. (2021). Adsorption of crystal violet dye using activated carbon of lemon wood and activated carbon/Fe_3_O_4_ magnetic nanocomposite from aqueous solutions: A kinetic, equilibrium and thermodynamic study. Molecules.

[16] Jiang Q., Chen Y., Yu S., Zhu R., Zhong C., Zou H., Gu L., He Q. (2020). Effects of citrus peel biochar on anaerobic co-digestion of food waste and sewage sludge and its direct interspecies electron transfer pathway study. Chem. Eng. J..

[17] Hao L., Zhang J., Liu J., Min Y., Chen C. (2023). Applications of Carbon-Based Materials in Activated Peroxymonosulfate for the Degradation of Organic Pollutants: A Review. Chem. Rec..

[18] Wan H., Wang F., Chen Y., Zhao Z., Zhang G., Dou M., Xue B. (2021). Enhanced Reactive Red 2 anaerobic degradation through improving electron transfer efficiency by nano-Fe_3_O_4_ modified granular activated carbon. Renew. Energy.

[19] Wang G., Li Q., Gao X., Wang X.C. (2018). Synergetic promotion of syntrophic methane production from anaerobic digestion of complex organic wastes by biochar: Performance and associated mechanisms. Bioresour. Technol..

[20] Zhao Z., Cao Y., Li S., Zhang Y. (2021). Effects of biowaste-derived biochar on the electron transport efficiency during anaerobic acid orange 7 removal. Bioresour. Technol..

[21] Lv L., Yin B., Zhang D., Ji W., Liang J., Liu X., Gao W., Sun L., Ren Z., Zhang G. (2024). Synchronous reinforcement azo dyes decolorization and anaerobic granular sludge stability by Fe, N co-modified biochar: Enhancement based on extracellular electron transfer. J. Hazard. Mater..

[22] Tong S., Chen D., Mao P., Jiang X., Sun A., Xu Z., Liu X., Shen J. (2022). Synthesis of magnetic hydrochar from Fenton sludge and sewage sludge for enhanced anaerobic decolorization of azo dye AO7. J. Hazard. Mater..

[23] Wang G.Y., Yang S.S., Ding J., Chen C.X., Zhong L., Ding L., Ma M., Sun G.S., Huang Z.L., Ren N.Q. (2021). Immobilized redox mediators on modified biochar and their role on azo dye biotransformation in anaerobic biological systems: Mechanisms, biodegradation pathway and theoretical calculation. Chem. Eng. J..

[24] Zhao X., Hu G., Chen G.F., Zhang H., Zhang S., Wang H. (2021). Comprehensive understanding of the thriving ambient electrochemical nitrogen reduction reaction. Adv. Mater..

[25] Zhang Z., Zhou Y., Zhang J., Xia S., Hermanowicz S.W. (2016). Effects of short-time aerobic digestion on extracellular polymeric substances and sludge features of waste activated sludge. Chem. Eng. J..

[26] Frølund B., Palmgren R., Keiding K., Nielsen P.H. (1996). Extraction of extracellular polymers from activated sludge using a cation exchange resin. Water Res..

[27] Zhao Z., Zhang Y., Yu Q., Dang Y., Li Y., Quan X. (2016). Communities stimulated with ethanol to perform direct interspecies electron transfer for syntrophic metabolism of propionate and butyrate. Water Res..

[28] Edgar R.C. (2013). UPARSE: Highly accurate OTU sequences from microbial amplicon reads. Nat. Methods.

[29] Quast C., Pruesse E., Yilmaz P., Gerken J., Schweer T., Yarza P., Peplies J., Glockner F.O. (2012). The SILVA ribosomal RNA gene database project: Improved data processing and web-based tools. Nucleic Acids Res..

[30] Ma P., Yin B., Wu M., Han M., Lv L., Li W., Zhang G., Ren Z. (2024). Synergistic enhancement of microbes-to-pollutants and inter-microbes electron transfer by Fe, N modified ordered mesoporous biochar in anaerobic digestion. J. Hazard. Mater..

[31] Lai C., Huang F., Zeng G., Huang D., Qin L., Cheng M., Zhang C., Li B., Yi H., Liu S. (2019). Fabrication of novel magnetic MnFe2O4/bio-char composite and heterogeneous photo-Fenton degradation of tetracycline in near neutral pH. Chemosphere.

[32] Wang Y.Q., Ding J., Pang J.W., Wu C.D., Sun H.J., Fang R., Ren N.Q., Yang S.S. (2024). Promotion of anaerobic biodegradation of azo dye RR2 by different biowaste-derived biochars: Characteristics and mechanism study by machine learning. Bioresour. Technol..

[33] Surendra B.S. (2018). Green engineered synthesis of Ag-doped CuFe_2_O_4_: Characterization, cyclic voltammetry and photocatalytic studies. J. Sci. Adv. Mater. Devices.

[34] Wang J., Yang Y., Bai L., Wang Y., Cui D., Zhao N., Zhao M. (2024). Efficient removal of Congo Red by carbonized Ganoderma lucidum spore with Fe_3_O_4_. J. Water Process Eng..

[35] Ma P., Jin M., Zhang D., Lv L., Zhang G., Ren Z. (2024). Surface engineering-based S, N co-doped biochar for improved anaerobic digestion: Enhancing microbial-pollutant and inter-microbial electron transfer synergistic EPS protection. J. Hazard. Mater..

[36] Che L., Xu H., Wei Z., Wei R., Yang B. (2022). Activated carbon modified with nano manganese dioxide triggered electron transport pathway changes for boosted anaerobic treatment of dyeing wastewater. Environ. Res..

[37] Nguyen T.H., Vo T.T., Watari T., Hatamoto M., Setiadi T., Yamaguchi T. (2024). Azo dye anaerobic treatment in anaerobic reactors coupled with PVA/Fe/Starch gel bead. Bioresour. Technol..

[38] Mohan S.V., Babu P.S., Srikanth S. (2013). Azo dye remediation in periodic discontinuous batch mode operation: Evaluation of metabolic shifts of the biocatalyst under aerobic, anaerobic and anoxic conditions. Sep. Purif. Technol..

[39] Zahran S.A., Ali-Tammam M., Hashem A.M., Aziz R.K., Ali A.E. (2019). Azoreductase activity of dye-decolorizing bacteria isolated from the human gut microbiota. Sci. Rep..

[40] Zhu Y., Xu J., Cao X., Cheng Y. (2018). Characterization of functional microbial communities involved in different transformation stages in a full-scale printing and dyeing wastewater treatment plant. Biochem. Eng. J..

[41] Li Y., Zhang Y., Liu Y., Zhao Z., Zhao Z., Liu S., Zhao H., Quan X. (2016). Enhancement of anaerobic methanogenesis at a short hydraulic retention time via bioelectrochemical enrichment of hydrogenotrophic methanogens. Bioresour. Technol..

[42] Park J.H., Kang H.J., Park K.H., Park H.D. (2018). Direct interspecies electron transfer via conductive materials: A perspective for anaerobic digestion applications. Bioresour. Technol..

[43] Xu H., Wang C., Yan K., Wu J., Zuo J., Wang K. (2016). Anaerobic granule-based biofilms formation reduces propionate accumulation under high H2 partial pressure using conductive carbon felt particles. Bioresour. Technol..

[44] Kato S., Hashimoto K., Watanabe K. (2012). Methanogenesis facilitated by electric syntrophy via (semi) conductive iron-oxide minerals. Environ. Microbiol..

[45] Hu Q., Sun D., Ma Y., Qiu B., Guo Z. (2017). Conductive polyaniline nanorods enhanced methane production from anaerobic wastewater treatment. Polymer.

[46] Wang Z., Zhang C., Watson J., Sharma B.K., Si B., Zhang Y. (2022). Adsorption or direct interspecies electron transfer? A comprehensive investigation of the role of biochar in anaerobic digestion of hydrothermal liquefaction aqueous phase. Chem. Eng. J..

[47] Li T., Yang X.L., Song H.L., Xu H., Chen Q.L. (2022). Quinones contained in wastewater as redox mediators for the synergistic removal of azo dye in microbial fuel cells. J. Environ. Manag..

[48] Song X., Liu J., Jiang Q., Zhang P., Shao Y., He W., Feng Y. (2019). Enhanced electron transfer and methane production from low-strength wastewater using a new granular activated carbon modified with nano-Fe_3_O_4_. Chem. Eng. J..

[49] Qin J., Qian L., Zhang J., Zheng Y., Shi J., Shen J., Ou C. (2021). Accelerated anaerobic biodecolorization of sulfonated azo dyes by magnetite nanoparticles as potential electron transfer mediators. Chemosphere.

[50] Barua S., Dhar B.R. (2017). Advances towards understanding and engineering direct interspecies electron transfer in anaerobic digestion. Bioresour. Technol..

[51] Wu Y., Wang S., Liang D., Li N. (2020). Conductive materials in anaerobic digestion: From mechanism to application. Bioresour. Technol..

[52] Shrestha P.M., Rotaru A.E. (2014). Plugging in or going wireless: Strategies for interspecies electron transfer. Front. Microbiol..

