# Supplementary material for: Rice Straw-Derived Magnetic Hydrothermal Carbon Accelerates Anaerobic Azo Dye Biodegradation Through Enhanced Interspecies Electron Transfer
Source: Biology (Basel). 2026 Jun 7;15(12):896. doi: 10.3390/biology15120896 (PMC13295486; doi:10.3390/biology15120896)
Supplement: Supplementary file 1 [file biology-15-00896-s001.zip › biology-4279615-supplementary.pdf]

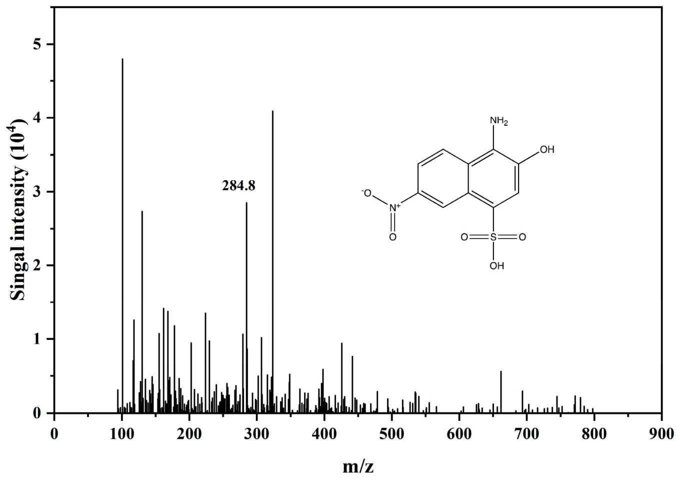

Figure S1 Identification of metabolite of Chrome Black T by LC-MS analysis.

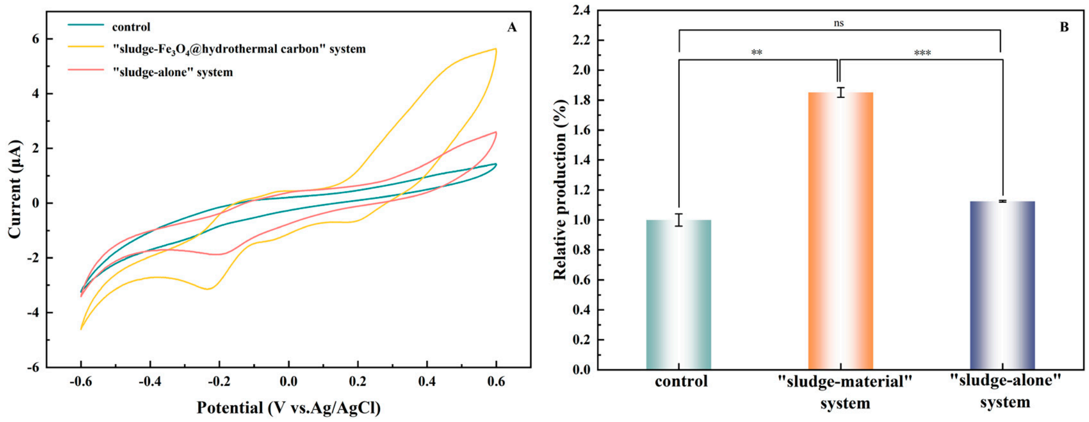

Figure S2 (A) Electrochemical analysis of EPS; (B) The relative production of cytochrome C within EPS in the different treatment systems.
